# Advances in biomaterials for sports injury prevention and rehabilitation: current status and future perspectives

**DOI:** 10.1039/d5na00874c

**Published:** 2025-12-30

**Authors:** Nan Zhang, Qinghua Meng, Miaomiao Xiao, Luxing Zhou, Hongshuai Leng, Chunyu Bao

**Affiliations:** a Tianjin University of Sport Tianjin 301617 China 202210700396@stu.tjus.edu.cn chunyubao730102@tjus.edu.cn; b Tianjin Sports Injury and Rehabilitation Virtual Simulation Experimental Teaching Center Tianjin 301617 China

## Abstract

This study focuses on sports injury materials in competitive sports and explores the potential of different types of materials in injury prevention, treatment and rehabilitation. As the level of competition increases and the intensity of training increases, the risk of injury to athletes increases and the demand for high performance protective materials increases. The development of new materials should not only meet the requirements of biomechanical adaptability and histocompatibility, but also have the characteristics of intelligent monitoring, rapid repair and personalised customisation, in order to enhance the effect of injury protection and accelerate the rehabilitation process. This paper analyses recent advances in polymer composites, nanomaterials and 3D printed materials and suggests that future developments should focus on the intelligence, personalisation and sustainability of materials. In addition, the study highlights the key role of interdisciplinary collaboration in advancing the application of materials for sports injuries and proposes improvement strategies to optimise the manufacturing process, enhance data analysis and improve biosafety to drive the field towards greater precision and efficiency.

## Abstract 2

This study focuses on sports injury materials in competitive sports and explores the potential of different types of materials in injury prevention, treatment and rehabilitation.

## Graphical abstract

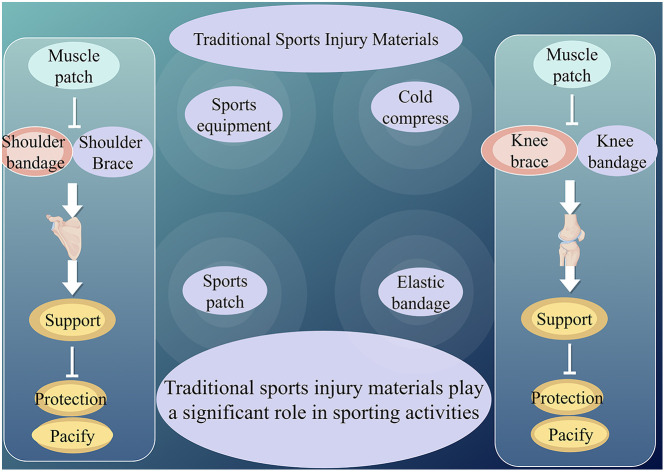

## 1. Introductory

Competitive sports, characterized by high intensity and rigorous challenges, have become an integral part of global sports culture.^1^ From the Olympic games to various world-class competitions, competitive sports continually push athletes to their limits, setting new records and redefining human potential^2^ However, the demands of high-intensity training and fierce competition have led to an increasing prevalence of sports injuries.^3–5^ Athletes in sprinting, basketball, football, and other disciplines are highly susceptible to a range of injuries, including muscle strains, joint sprains, fractures, and soft tissue damage.^6–10^ Such injuries not only compromise an athlete's physical well-being but can also disrupt training schedules and, in severe cases, prematurely end professional careers. Consequently, the effective prevention and treatment of sports injuries have become pivotal issues in the field of sports medicine.

Traditional approaches to managing sports injuries, such as physical therapy, pharmaceutical interventions, surgical procedures, and rehabilitation training, have been widely employed in sports medicine.^11^ Physical therapy techniques—including cold and heat therapy, electrical stimulation, ultrasound, and laser therapy—can provide temporary pain relief and improve blood circulation, but they often fail to facilitate long-term tissue repair.^12^ Pharmaceutical treatments, such as nonsteroidal anti-inflammatory drugs (NSAIDs), effectively alleviate inflammation and pain; however, prolonged use may lead to side effects such as gastrointestinal discomfort and kidney dysfunction, with limited benefits for tissue regeneration.^13^ For severe injuries such as ligament tears or fractures, surgical interventions—including arthroscopic surgery, ligament reconstruction, and bone grafting—remain the primary treatment options. Nevertheless, these procedures are often invasive, involve prolonged recovery periods, and yield varied rehabilitation outcomes due to individual differences.^14^ Additionally, rehabilitation training, such as functional and progressive load training, plays a critical role in restoring an athlete's mobility. However, given the high frequency and intensity of competitive sports, complete prevention of reinjury remains challenging.^16^ Overall, traditional treatment methods exhibit certain limitations, including restricted applicability, outcome variability among individuals, extended recovery timelines, and an inability to meet the demands of elite athletes seeking rapid return to competition. This underscores the urgent need for more advanced protective and therapeutic strategies for sports injuries.

With rapid advancements in materials science and biomedical technology, innovative sports injury materials have emerged as a research hotspot in competitive sports.^16^ Modern polymeric materials, smart materials, and nanomaterials have been extensively applied in protective gear, rehabilitation equipment, implantable repair materials, and intelligent monitoring systems.^17–20^ These materials not only enhance physical protection and reduce injury risks but also accelerate tissue repair and improve rehabilitation efficiency.^21^ For instance, nanofiber scaffolds, biomimetic photothermal nanomaterials, and intelligent hydrogels have demonstrated significant potential in sports injury treatment and recovery. Investigating the application of these materials in competitive sports not only helps summarize the latest technological advancements but also provides a theoretical foundation and practical guidance for future material innovations. Sports biomaterials can be broadly classified into preventive materials, which aim to reduce injury risk, and therapeutic materials, which promote tissue repair and rehabilitation.

This paper first categorizes and discusses common sports injuries in competitive sports, followed by an analysis of the classification and current applications of sports injury materials. The study then explores recent technological advancements and innovations in this field. Finally, through a comprehensive review of the application landscape of sports injury materials in competitive sports, we highlight the strengths and limitations of existing technologies and provide insights into future developments. Advancing high-performance sports injury materials is not only essential for safeguarding athletes health and careers but also plays a crucial role in the sustainable development of competitive sports and the enhancement of overall athletic performance.

## 2. Classification and common types of sports injuries in competitive sports

Competitive sports are characterized by high intensity and frequent physical confrontations, making athletes susceptible to various types of sports injuries during training and competition.^3^ Such injuries not only hinder athletic performance but may also lead to long-term health complications.^4^ Therefore, a comprehensive understanding of the common types of sports injuries, their underlying mechanisms, and influencing factors is essential for developing more effective prevention and rehabilitation strategies.

Sports injuries in competitive sports can be classified based on different criteria, allowing for a better understanding of their occurrence mechanisms, clinical manifestations, and corresponding treatment and prevention measures.^22^ The two most common classification approaches are mechanism-based classification and tissue-type classification.

### 2.1 Mechanism-based classification: acute *vs.* chronic injuries

Based on the mechanism of occurrence, sports injuries can be categorized into acute injuries and chronic injuries.^23^ Sports injuries can also be classified based on the type of tissue affected, broadly categorized into soft tissue injuries and bone injuries.^24^

#### 2.1.1 Acute injuries

Acute injuries occur suddenly due to an external force or excessive stretching of tissues during movement. They are typically characterized by an abrupt onset, intense pain, and visible tissue damage.^25^ These injuries are prevalent in high-impact and contact sports such as football, basketball, and rugby. Common types of acute injuries include fractures, dislocations, ligament tears, muscle strains, and joint sprains.^26^

For instance, in basketball, a player landing improperly after a jump may excessively twist their ankle, resulting in a sprained ankle or ligament tear. Similarly, in football, sudden stops and directional changes can cause an anterior cruciate ligament (ACL) tear.^27^ Typical symptoms of acute injuries include severe pain, swelling, subcutaneous bruising, and restricted movement. Severe cases may require surgical intervention and long-term rehabilitation.^28^

The RICE protocol (Rest, Ice, Compression, Elevation) is commonly used for the immediate management of acute injuries, helping to reduce inflammation and pain. Further rehabilitation treatment is prescribed based on the severity of the injury.^29^

#### 2.1.2 Chronic injuries

Chronic injuries result from prolonged, repetitive, or excessive use of a specific body part. These injuries develop gradually, often with mild symptoms in the early stages that worsen over time.^30^ Chronic injuries are more common in sports requiring repetitive movements or sustained high-intensity training, such as long-distance running, swimming, tennis, and weightlifting.

Common chronic injuries include tendinitis (*e.g.*, Achilles tendinitis, tennis elbow), stress fractures, arthritis, cartilage wear, and shin splints.^31^ For example, long-distance runners who train excessively without adequate recovery may develop stress fractures in the tibia, while tennis players frequently using their arms for swinging motions are prone to tennis elbow (lateral epicondylitis).^32^

The primary symptoms of chronic injuries include persistent pain, stiffness, localized inflammation, and functional limitations. Without timely intervention, these injuries may become irreversible.^33^ Preventative and therapeutic measures for chronic injuries focus on proper training volume management, strength conditioning, recovery optimization, and early intervention through physical therapy, training modifications, and supportive gear.^34^

### 2.2 Tissue-based classification: soft tissue *vs.* bone injuries

#### 2.2.1 Soft tissue injuries

Soft tissue injuries refer to damage to muscles, tendons, ligaments, skin, blood vessels, and nerves, often resulting from direct impact, excessive stretching, or repetitive use. These injuries are common across various competitive sports.^35^ Based on the type of damage, soft tissue injuries include contusions, strains, sprains, tendinitis, and bursitis.^36^ In football and basketball, muscle contusions often occur due to direct collisions between players, leading to localized swelling, subcutaneous bruising, and tenderness. Sprinters may suffer from hamstring strains due to excessive force exertion at the start of a race, causing muscle fiber tears and movement restrictions.^37^ In racket sports such as tennis and badminton, repeated arm movements increase the risk of tendinitis, which manifests as chronic pain and localized inflammation. Recovery time for soft tissue injuries varies depending on severity. Mild injuries can often be managed with the RICE protocol, whereas severe injuries may require rehabilitation training or medical intervention.^29^ Scientific training, thorough warm-ups, and adequate recovery are crucial for reducing the risk of soft tissue injuries.

#### 2.2.2 Bone injuries

Bone injuries result from external trauma, overuse, or structural weaknesses in the skeletal system. The most common types of bone injuries include fractures, stress fractures, and periostitis.^38^ Fractures occur due to high-impact forces, falls, or excessive torsion. For instance, football players falling during tackles may sustain clavicle fractures, while basketball players landing improperly might suffer from tibia fractures.^39^ Stress fractures develop from prolonged repetitive stress on bones, commonly seen in endurance athletes like marathon runners. These injuries present as microcracks in the bone, accompanied by chronic pain. Periostitis, an inflammation of the periosteum (the tissue surrounding bones), is caused by excessive strain on bones, such as shin splints in long-distance runners and long jumpers.^40^ Bone injuries often result in severe pain, swelling, and functional limitations, with serious cases requiring surgical fixation. Preventive strategies include scientific training plans, controlled load management, and adequate nutritional intake (*e.g.*, calcium and vitamin D supplementation) to enhance bone density and reduce injury risks.^41^ By systematically understanding the classification and characteristics of sports injuries in competitive sports, athletes, coaches, and medical professionals can adopt more effective injury prevention and treatment strategies, ultimately enhancing athletic performance and ensuring long-term physical well-being.

## 3. Classification and application status of sports injury materials

The application of sports injury materials in competitive sports is crucial, and they are not only used to prevent sports injuries, but also to assist treatment and rehabilitation. According to their purpose and functional characteristics, sports injury materials can be broadly divided into two main categories: (1) preventive materials and devices, and (2) therapeutic and rehabilitative materials and devices.

With the continuous development of materials science, new sports injury materials are constantly optimised in terms of protection, functionality and comfort, improving the safety and competitive performance of athletes. According to the use and function of materials, sports injury materials can be classified into traditional injury protection materials and modern high-tech sports injury materials (see Table 1 for a comparison of the two types of materials).

**Table 1:** Comparison of traditional injury materials and modern high-tech sports injury materials

Comparison dimension	Traditional sports injury materials	Modern high-tech materials for sports injuries
Material type	Cotton, leather, elastic bandages, silicone	Nanomaterials, polymer composites, smart materials, 3D printing materials
Protective properties	Primarily provides basic cushioning, support, and immobilisation but limited protection	With high-strength energy absorption, intelligent monitoring, precise support, and stronger protective effect
Comfort	Often thicker and heavier, with average breathability and comfort	Lightweight design, breathable and ergonomically optimised
Adaptability	Fixed size, cannot be personalised	Individual adaptations available through 3D printing and intelligent adjustment
Durability	Easy to wear and tear, loss of support after prolonged use	Strong durability, the material can maintain stability for a long time
Rehabilitation functions	Mainly plays a passive support and immobilisation role	Can be combined with biomaterials, photothermal therapy, to promote tissue self-repair
Technological intelligence	No intelligent function, only relies on physical support	With intelligent sensing, pressure feedback, data monitoring, real-time analysis of the state of the movement
Representative products	Traditional protective gear (knee pads, wrist pads), ice packs, bandages	Nano brace, smart compression brace, 3D printed rehabilitation brace, graphene sportswear
Application scenarios	Suitable for general sports protection and basic injury treatment	For high-intensity athletics, precision protection, smart rehabilitation

### 3.1 Traditional injury protection materials and their current applications

Traditional sports injury protection materials hold a significant position in competitive sports. These materials are typically characterized by their simplicity, convenience, and affordability, making them suitable for most athletes in their daily training, competitions, and rehabilitation processes.^42^ The primary functions of these materials include injury prevention, pain relief, and accelerated recovery. Over time, they have been proven effective in supporting athletic performance and reducing injury risks.^43^ The most commonly used traditional sports injury materials include protective gear, cold and hot therapy materials, elastic bandages, and kinesiology tapes.^44^

#### 3.1.1 Protective gear

Protective gear is one of the most widely used traditional sports injury materials. Its main purpose is to support, protect, and stabilize joints, preventing sprains, strains, or other joint injuries during high-intensity training and competitions.^45^ For example, knee pads, wrist guards, and elbow pads are essential equipment for athletes in sports such as basketball, soccer, and skiing. These protective gears effectively reduce joint stress and absorb external impacts, thereby lowering the risk of injury.^46^ In extreme sports, helmets and back protectors play a crucial role in minimizing the risk of head and spinal injuries.

#### 3.1.2 Cold and hot therapy materials

Cold and hot therapy materials are classic choices for treating sports injuries, commonly used for both acute and chronic injury management.^47^ Ice packs and cooling gels are typically applied immediately after an acute injury to reduce inflammation and control swelling by lowering the temperature of the affected area. For example, in cases of ankle sprains, cold therapy helps in rapidly relieving swelling and alleviating pain.

Conversely, heat packs and therapeutic heat patches are used during the recovery phase of chronic injuries to promote blood circulation, relieve muscle stiffness, and relax tense muscles. They are commonly used to alleviate issues such as lower back muscle fatigue and knee arthritis.^48^

#### 3.1.3 Elastic bandages and kinesiology tape

Elastic bandages and kinesiology tapes serve as essential stabilization and support materials, helping in injury prevention and rehabilitation acceleration. Elastic bandages provide firm compression around injured areas, limiting joint movement to prevent further damage. They are commonly used in cases of ankle sprains and knee ligament injuries.^49^

Kinesiology tape, with its unique elasticity and adherence, not only reduces muscle fatigue and enhances athletic performance but also offers extra support and stabilization for muscles and joints during movement. Athletes suffering from tennis elbow and runner's knee frequently use kinesiology tape to prevent and mitigate injuries.^50^

Due to their practicality, affordability, and effectiveness, these traditional injury protection materials are widely applied across various sports. Whether for preventive protection (such as protective gear and bandages) or rehabilitative treatment (such as cold and hot therapy), they remain an indispensable part of an athlete's training and competition regimen.

Although modern materials and technologies continue to advance, traditional sports injury materials remain fundamental and essential. They not only help athletes extend their careers but also play a crucial role in injury relief and rehabilitation when injuries occur.^42^

#### 3.1.4 Illustrating traditional injury protection materials through the Chinese mythological figure Sun Wukong

**Fig. 1:**
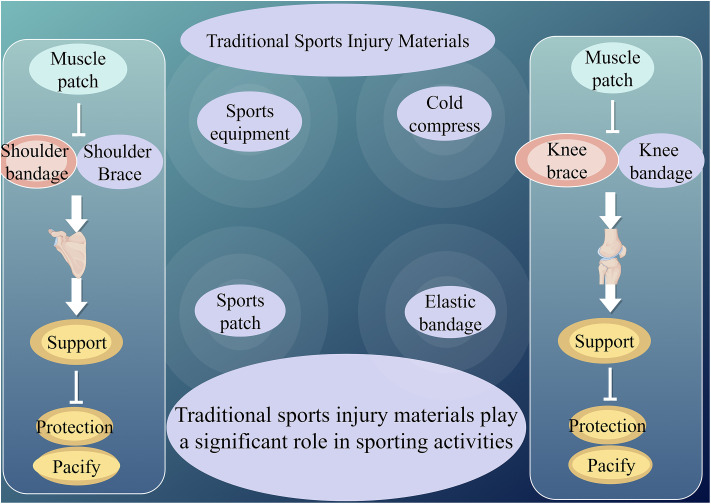
Application of traditional sports injury materials.

To further illustrate the characteristics of traditional injury protection materials (Fig. 1), we analyze the legendary Chinese mythological figure, Sun Wukong (the Monkey King), and his battle attire. In traditional martial arts and combat scenarios, Sun Wukong's equipment was primarily made from natural materials and simple craftsmanship, reflecting the features of early injury protection materials.

#### 3.1.5 Golden headband and restraints

Sun Wukong's golden headband symbolizes ancient methods of injury protection through simple restraints, akin to early bandages or wraps. While these restraints provided some level of stabilization and support, they lacked adjustability and often restricted blood circulation in localized areas.

#### 3.1.6 Beast hide armor and rattan armor

His battle attire, likely crafted from beast hide, hemp cloth, or rattan armor, resembled early protective gear such as leather knee pads and fabric wrist guards. Although these materials provided some degree of protection, they lacked sufficient cushioning and were prone to wear and tear during intense activity.

#### 3.1.7 Iron protective gear and weapons

Sun Wukong's golden cudgel, made of metal, was highly durable but lacked the lightweight and high-toughness characteristics of modern alloys. Similarly, ancient injury protection materials often included iron-based gear, such as iron shin guards and iron helmets, which provided rigid protection but significantly reduced mobility due to their weight.

#### 3.1.8 Herbal remedies and traditional therapies

After sustaining injuries in battle, Sun Wukong relied on elixirs and herbal medicine to recover. This reflects traditional sports injury treatments such as herbal poultices and heat therapy, which, although somewhat effective, often required long recovery times and lacked the precision and controlled effectiveness of modern medical materials.

The limitations of these traditional protective methods have driven the evolution of modern sports injury protection materials. Today, advancements in material science and technology provide athletes with lighter, more efficient, and more protective gear. However, traditional materials remain an integral part of sports injury prevention and rehabilitation, having laid the foundation for modern sports medicine while continuing to serve as essential components in athletic training and injury management.^42^

### 3.2 Modern high-tech sports injury materials and their applications

With rapid advancements in materials science and technology, modern high-tech sports injury materials have evolved far beyond basic protection and support functions. These materials now incorporate features such as intelligence, personalization, and responsiveness, offering enhanced injury prevention, repair, and rehabilitation. Innovations in nanotechnology, smart materials, and 3D printing have revolutionized the field of sports injury protection.^51^ The primary categories of modern high-tech sports injury materials include nanomaterials, polymer composites, and 3D-printed materials.

#### 3.2.1 Nanomaterials in sports injury prevention and rehabilitation

With the development of competitive sports, materials for sports injury protection and rehabilitation have evolved from traditional materials to modern high-tech materials, among which nanomaterials have shown great potential for sports injury monitoring, repair and regeneration. With their unique physical, chemical and biological properties, nanomaterials are driving the development of sports injury materials towards intelligence, personalisation and sustainability, and their applications cover a wide range of areas including sports monitoring, bone and cartilage repair, cytoskeletal modulation and energy harvesting.

##### 3.2.1.1 Application of nanomaterials in sports injury repair

**Fig. 2:**
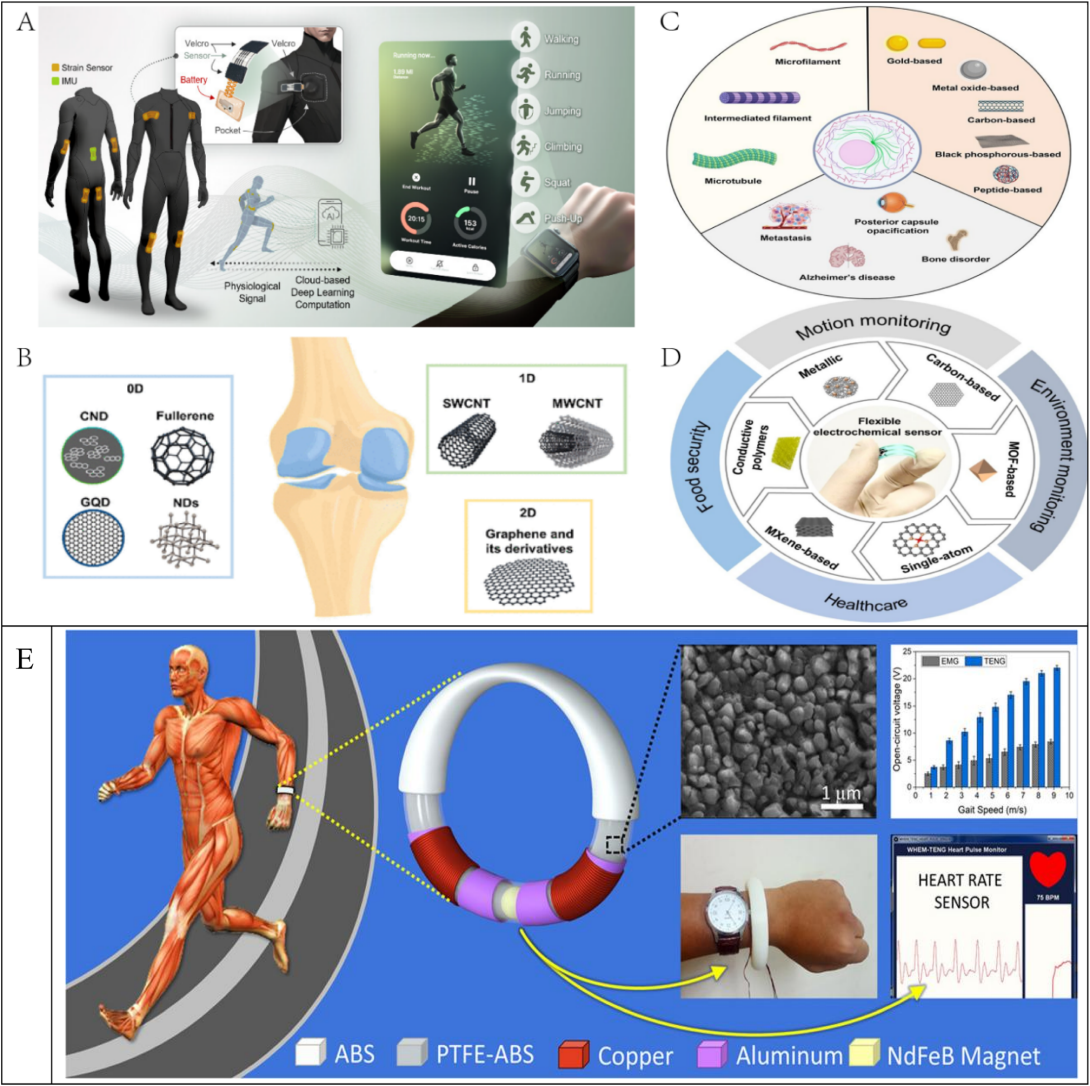
Application of nanomaterials in competitive sports ((A) reproduced from ref. 66 with permission from the American Chemical Society, copyright 2025; (B) reproduced from ref. 61 with permission from the American Chemical Society, copyright 2021; (C) reproduced from ref. 56 with permission from Elsevier, copyright 2024; (D) reproduced from ref. 68 with permission from Elsevier, copyright 2025; (E) reproduced from ref. 67 with permission from Elsevier, copyright 2018).

Bone and cartilage injuries in sports are extremely common, and traditional repair methods can have side effects due to large individual differences.^11–15^ Nanomaterials, especially carbon-based nanomaterials, have shown excellent biocompatibility and mechanical properties in bone, cartilage and osteochondral repair.^52^ Zero-dimensional nanomaterials can promote osteoblast proliferation and accelerate bone tissue regeneration;^53^ one-dimensional carbon nanotubes can be used to enhance the stability of bone repair scaffolds due to their excellent mechanical properties;^54^ and two-dimensional graphene materials can mimic the natural bone matrix and improve the regeneration efficiency of bone and cartilage tissue.^55^ The use of these materials not only improves the repair of sports injuries, but also reduces the risk of post-operative complications, allowing athletes to return to training more quickly (see C in Fig. 2).^56^

Carbon nanofibre composites and nanoceramic coatings provide enhanced impact resistance and are effective in reducing collision-related joint injuries in high-contact sports (*e.g.* football, basketball, rugby).^57^ In addition, the nanosilver coating has antimicrobial and anti-inflammatory properties that can be used to reduce skin infections caused by prolonged wear of protective equipment and improve athlete comfort.^58^ Sports ligament injuries are caused by overuse, improper training or external impact and are particularly common in high-impact sports such as basketball and football; nanofibre scaffolds have been widely investigated for ligament protection and repair due to their good biocompatibility and mechanical strength.^59^ For example, filipin protein–polycaprolactone nanofibrous membranes have been experimentally shown to have higher cell adhesion, biocompatibility and ligament repair ability than conventional materials, which can effectively increase the load-bearing capacity of ligaments and thus reduce the incidence of sports injuries.^60^

##### 3.2.1.2 Nanomaterials in cytoskeletal regulation

Recovery from sports injuries involves remodelling of cellular tissues, and the cytoskeleton plays a key role in this process. Nanomaterials have been shown to be able to promote repair of damaged tissues by influencing the stability and dynamic regulation of the cytoskeleton (see B in Fig. 2). Magnetic field-responsive and light-responsive nanomaterials can manipulate the cytoskeleton in a targeted manner, improving the precision of cell proliferation and tissue regeneration. In addition, inorganic nanomaterials (*e.g.* gold, metal oxides, black phosphorus) and organic nanomaterials (*e.g.* peptides and proteins) are emerging as a new generation of cytoskeletal modulation tools, offering the possibility of personalised treatment of sports injuries.^61^

In recent years, bionic photothermal nanomaterials have shown great potential in the prevention and treatment of sports injuries. These materials are based on the natural bionic principle of converting light energy into heat energy through photothermal conversion factors, promoting blood circulation in local tissues and improving the repair ability of damaged tissues.^62^ For example, HA–CuS gel combined with near-infrared photothermal therapy technology can significantly reduce inflammation and accelerate the recovery of damaged tissue in a short period of time, providing athletes with a more effective means of injury prevention.^63^ Cartilage damage in the knee is a relatively common injury in competitive sports, particularly during prolonged running, jumping or rapid turning movements. Nanostructured materials, such as rosette carbon nanotubes and PLA composite scaffolds, play a key role in cartilage tissue engineering.^64^ Studies have shown that the scaffold can promote the differentiation of bone marrow mesenchymal stem cells into chondrocytes and enhance the regenerative capacity of cartilage tissue, thereby improving joint tolerance and effectively reducing exercise-induced cartilage injury. Meanwhile, early detection of sports injuries is crucial to prevent further injury.^65^

##### 3.2.1.3 Nanomaterials in motion monitoring

Traditional motion monitoring devices are often limited to single-point data collection, making it difficult to achieve real-time detection across the entire body. The introduction of nanomaterials, on the other hand, allows flexible electronic sensors to be combined with smart textiles to form highly sensitive wearable motion detection systems (see A in Fig. 2).^66^ For example, a wireless monitoring kit based on nanomembranes and laser-induced graphene strain sensors is able to accurately track an athlete's whole-body motion status and analyse the motion data using deep learning techniques to predict a variety of motion types, including running, jumping and push-ups, with an accuracy rate of 95.3%. The combination of such nanomaterial sensors provides advanced technical support for personalised sports health management and injury warning. Competitive sports have placed higher demands on long-term sports monitoring and data collection, while traditional wearable devices rely on battery power and have problems such as short battery life and inconvenient charging. To address this challenge, researchers have developed the Curvilinear Wearable Hybrid Electromagnetic Friction Nanogenerator (WHEM-TENG), a 3D-printed nano-energy harvesting device for efficient energy harvesting during exercise (see E in Fig. 2).^67^ The device can continuously power an electronic watch for 410 seconds after 5 seconds of running; it can also power a self-powered heart rate sensor for continuous monitoring of the athlete's physiological state. This technological breakthrough indicates that nanomaterials have a broad application prospect in wearable energy harvesting, which is expected to promote the development of self-powered smart sports equipment and make sports monitoring more convenient and efficient. Relevant researchers on nanomaterial flexible electrochemical sensors have reviewed various applications in sports monitoring, environmental monitoring, medical diagnostics, and food quality and safety, while discussing the challenges and future directions of flexible electrochemical sensing (see D in Fig. 2).^68^

Nanomagnetic materials combined with biosensor technology can be used to construct highly sensitive immunosensors for real-time monitoring of knee, ankle and tendon injuries in athletes.^69^ For example, in the study of medial collateral ligament injuries in the knee joint of football players, nanomagnetic materials can detect training load, injury risk and recovery status, and provide personalised training and rehabilitation advice to athletes, effectively reducing the incidence of injury. The speed of recovery from sports injuries directly affects the competitive status of athletes, and nanomaterials play an important role in rehabilitation medicine.^70^ For example, multifunctional nanomaterial particles can be used as drug carriers to deliver anti-inflammatory drugs to the injured area, thereby improving therapeutic efficacy and reducing drug side effects. Experimental studies have shown that the use of drug-loaded nanomaterial particles to treat sports injuries results in significantly better recovery than traditional treatments and reduces the risk of injury recurrence. The application of nanomaterials is driving changes in the field of injury prevention in competitive sports.^71^ From high-performance protective gear, bionic photothermal materials, nanofibre ligament scaffolds to smart biosensors, nanomaterials not only provide more advanced injury protection solutions, but also reduce the incidence of sports injuries and accelerate the recovery process through accurate detection and efficient treatment.

The introduction of nanomaterials has greatly enhanced the intelligence and precision of sports injury protection materials, showing revolutionary application value in the fields of sports monitoring, injury repair, cell regulation and self-powered devices. In the future, with the integration of nanotechnology, biomedical engineering and artificial intelligence, sports injury protection and rehabilitation materials will be more efficient, personalised and sustainable, providing athletes with more comprehensive safety protection and scientific training support.

#### 3.2.2 Polymer composites

Polymer composites have become an important research direction in the field of sports injury prevention due to their light weight, high strength, impact resistance and good biocompatibility.^72^

##### 3.2.2.1 Application of polymer composites in sports injury repair

Polymer composites are widely used in sports equipment, protective gear, rehabilitation equipment and biological implants, which can effectively reduce the incidence of sports injuries and improve the safety and competitive performance of athletes. Sports protective gear is an important means of reducing injuries in competitive sports, and the introduction of polymer composites has significantly improved the durability, cushioning and comfort of protective gear.^73^ For example, Ni–SiC nanocomposites have been used as surface coatings for sports equipment such as dumbbells to improve their wear resistance through pulsed electrodeposition technology, thereby reducing the risk of injury to athletes due to wear or breakage of the equipment during use.^74^ In addition, polyetheretherketone composites are used in orthopaedic implants such as artificial joints and ligament scaffolds due to their excellent mechanical strength and biocompatibility, which help athletes recover faster from injuries and reduce post-operative complications; graphene composites show great potential for sports injury prevention due to their ultra-high strength, excellent electrical conductivity and lightweight properties. In the study of internal fixation of ankle fractures in sports, graphene composites are used to enhance the strength, stability and biocompatibility of the fixation device, which effectively reduces secondary injuries caused by joint instability in athletes. Meanwhile, graphene-rubber composites can be used in the soles of high-performance sports shoes to enhance the cushioning and shock-absorbing effect, thus reducing the impact of prolonged exercise on the knee and ankle joints.^75^ In the design of sports shoes, the addition of hollow glass microspheres to ethylene vinyl acetate foam can significantly improve the shock absorption capacity and abrasion resistance of the sole, reduce the impact of running and jumping on the joints, and further optimise the protective effect against sports injuries.^76^

**Fig. 3:**
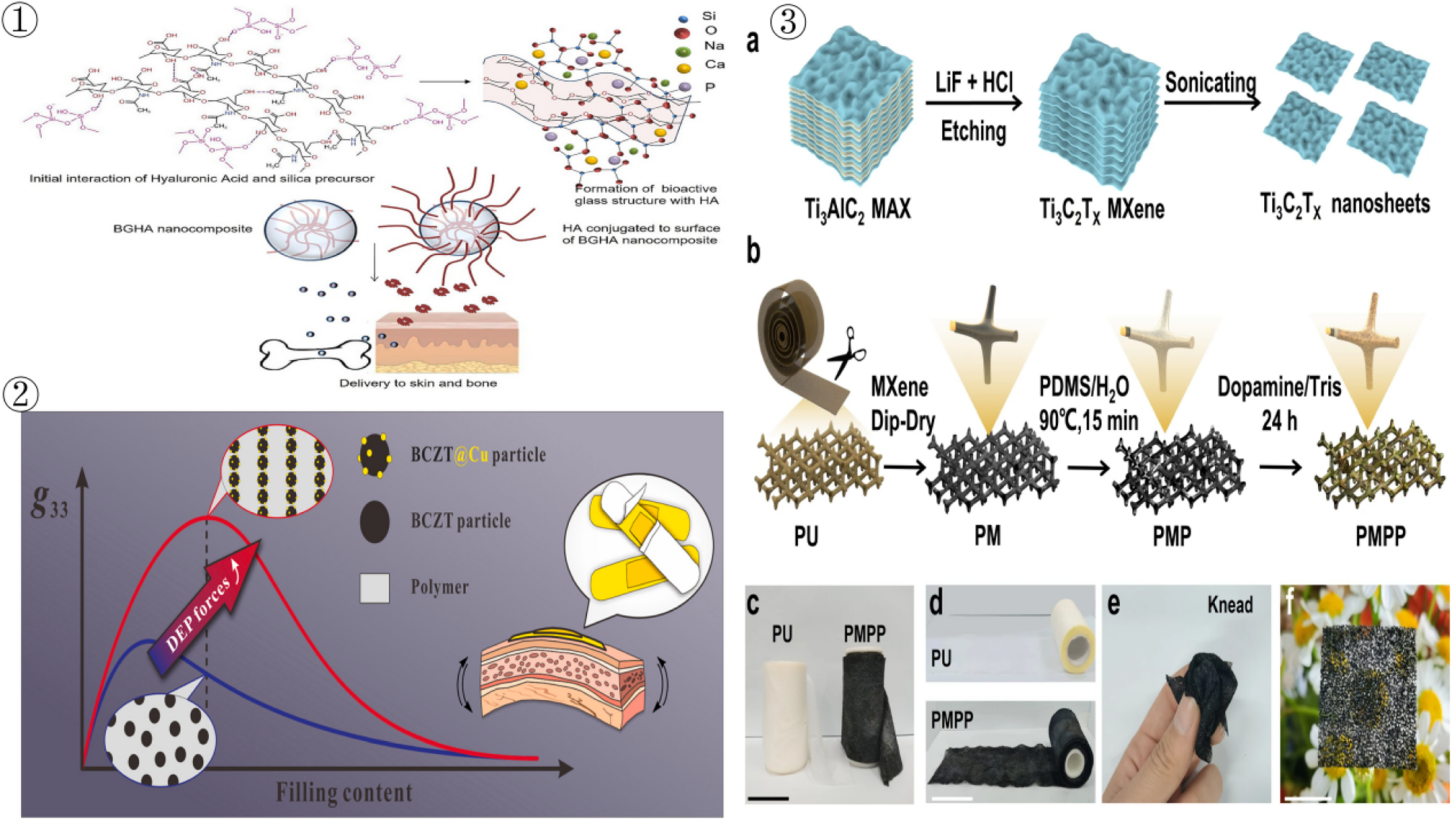
Application of polymer composites in competitive sports (① reproduced from ref. 77 with permission from Elsevier, copyright 2023; ② reproduced from ref. 81 with permission from Springer Nature, copyright 2022; ③ reproduced from ref. 82 with permission from Wiley, copyright 2022).

Bioactive glass (BG) and hyaluronic acid (HA) nanocomposites (BGHA) have been shown to be effective in delivering hyaluronic acid to skin and bone (see ① in Fig. 3), providing a new solution for non-invasive treatments such as osteoarthritis, sports injury treatment and wound healing.^77^ Its features include improved biomimetic synthesis methods to avoid the use of harmful chemicals and improve biosafety. The high biocompatibility of the nanoparticles results in skin and bone cell survival rates of over 70% and cellular uptake rates of 90–100%. The composites can penetrate the skin barrier and are expected to be used in sports injury repair, eye drops, dermal fillers, *etc.* The presence of BG enhances bone tissue regeneration, further extending the therapeutic applications for sports injuries. In addition, this nanocomposite can be widely used in cosmeceutical products such as moisturisers, anti-wrinkle creams and shampoos, and has the same potential for use in skin care after sports injuries. Three-dimensional bone scaffolds made of polycaprolactone combined with nanohydroxyapatite and tricalcium phosphate can promote bone formation, improve the mechanical strength of bones and accelerate the rehabilitation process of athletes after fractures.^78^ Meanwhile, orthodontic coupler devices combined with polymer composites have demonstrated improved stability and biomechanical properties in tendon repair, significantly reduced postoperative complications, and accelerated tendon regeneration.^79^ In terms of dental protection against sports injuries, zirconia-reinforced lithium silicate and titanium-zirconia composites have better biomechanical properties and pressure distribution ability than conventional implants, which can effectively reduce the incidence of dental injuries.^80^

##### 3.2.2.2 Application of piezoelectric composites in motion monitoring and injury warning

Piezoelectric composites (PCs) based on dielectric electrophoresis (DEP) technology were used to monitor the movement and injury warning of Ba_0_._85_Ca_0_._15_Ti_0_._9_Zr_0_._1_O_3_ (BCZT) particles decorated with Cu nanoparticles, which greatly improves the piezoelectric properties of the material (see ② in Fig. 3). The features include an ultra-high piezoelectric voltage constant (*g*_33_ = 720 × 10^−3^ V m N^−1^), which far exceeds that of similar materials and improves the sensitivity of motion monitoring. The prepared flexible sensors can seamlessly fit the human body, especially for joints, and are as comfortable and stable as a band-aid.^81^ The sports health monitoring system developed in conjunction with this material enables real-time warning of dangerous movements, correction of incorrect postures, and prevention of sports injuries, thus reducing the risk of chronic injuries caused by improper postures in athletes. The application of this novel composite material makes injury prevention and rehabilitation monitoring in competitive sports training more efficient and accurate.

##### 3.2.2.3 Multifunctional composites in intelligent rehabilitation and therapy

With the popularity of smart wearable devices, multifunctional composites that integrate highly sensitive sensing, rehabilitation therapy and health monitoring are becoming one of the key technologies for future sports injury prevention. For example, MXene/PDMS/PDA/PU nanocomposites exhibit excellent flexibility, breathability and multifunctionality for sports detection and injury treatment (see (iii) in Fig. 3).^82^ Features include excellent flexibility and breathability for prolonged wear and reduced discomfort for athletes. Photothermal therapy with antimicrobial activity, which can be applied directly to the site of sports injuries for thermal therapy to speed up injury recovery and prevent infection. Combined with the wireless smart insole and cushion, real-time monitoring of gait, sitting posture and other data can be used to assess the progress of sports injury rehabilitation and provide postural correction recommendations. The application of this composite material can not only play a role in the detection and treatment of sports injuries, but also promote the development of wearable medical devices, providing more opportunities for sports rehabilitation.

Overall, polymer composites are playing an increasingly important role in the prevention, monitoring and treatment of competitive sports injuries due to their excellent mechanical properties, light weight, durability and biocompatibility. In the future, with the deep integration of materials science and intelligent technology, polymer composites will show greater potential in personalised protection, real-time monitoring and rapid rehabilitation of sports injuries, thus providing greater support for the development of competitive sports.

#### 3.2.3 3D printing materials

With the development of 3D printing technology, its application in competitive sports is gradually expanding to the prevention of sports injuries, rehabilitation and personalised medicine.^83^ 3D printing materials have been widely used in sports protectors, orthopaedic devices, orthopaedic implants and rehabilitation equipment.^84^

##### 3.2.3.1 3D printing in orthopaedic treatment of sports injuries

In the field of sports protective equipment, 3D printed polymer materials (*e.g.* polyurethane, polylactic acid, polyetheretherketone) can be used to create ergonomic knee and ankle pads and helmets based on the biomechanical properties of individual athletes, providing more precise support and protection and reducing joint and tendon injuries.^85^ In addition, 3D-printed finger orthoses based on a new thermoplastic polyurethane material offer superior stiffness and toughness to ensure that athletes' fingers are adequately immobilised during the rehabilitation process, and wireless sensors can be integrated to monitor recovery in real time.^86^ For patients with bone and joint injuries, 3D-printed bone and joint implants and surgical guides can improve the accuracy of pre-operative planning, reduce post-operative complications and improve the efficiency of athletes' post-operative recovery.^87^ For example, in the treatment of tibial plateau fracture deformity, 3D-printed surgical navigation models can improve the accuracy of osteotomies, reduce intraoperative blood loss and shorten operative time, significantly improving patients' postoperative functional recovery.^88^

3D printing technology also has great potential in the repair of cartilage and ligament injuries.^89^ Due to the weak self-repair ability of cartilage after injury, scientists have developed 3D-printed scaffolds based on sodium alginate-gelatin composite hydrogel, which can promote chondrocyte proliferation and improve repair efficiency.^90^ In addition, low-temperature 3D printing of bionic biphasic scaffolds can be used to produce tissue-engineered scaffolds that mimic the heterogeneity of natural osteochondral cartilage, thereby accelerating the healing process after osteoarticular injuries.^91^ For knee meniscus injury, researchers have developed tissue-engineered meniscus with gradient-sized diamond pore microstructure, which can successfully induce meniscus regeneration and effectively alleviate joint degeneration in rabbit knee joint experiments.^92^ In addition, 3D-printed flexible electronic devices, such as conductive hydrogel-based smart sensors, can be seamlessly attached to athletes' joints to achieve sports posture monitoring and injury warning, providing real-time feedback to athletes to optimise training methods and reduce injuries caused by incorrect posture.^93^ In conclusion, 3D printing technology has a broad application prospect in the prevention, rehabilitation and treatment of competitive sports injuries, and its application in the field of sports injury prevention will become more accurate and efficient in the future with the development of smart materials, personalised medicine and bionic structure optimisation.

##### 3.2.3.2 3D printing in sports injury rehabilitation and prosthesis design

**Fig. 4:**
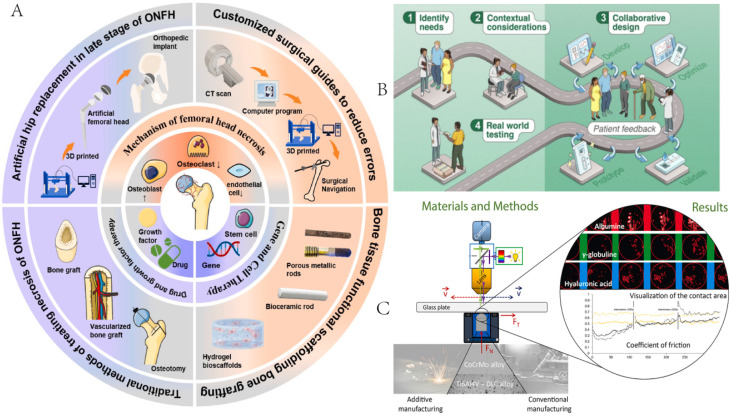
3D printing materials in competitive sports ((A) reproduced from ref. 97 with permission from Elsevier, copyright 2025; (B) reproduced from ref. 103 with permission from the American Chemical Society, copyright 2025; (C) reproduced from ref. 98 with permission from Elsevier, copyright 2022).

Athletes are susceptible to orthopaedic injuries such as fractures, osteonecrosis (ONFH), joint injuries and other injuries during high-intensity training and competition, and 3D printing technology offers unique advantages in these areas: personalised implants: unlike traditional hip replacement surgery, 3D printing allows precise positioning of the necrotic area and creation of a personalised implant, which is particularly suitable for hip preservation therapy in young athletes.^94,95^ Bone tissue engineered scaffolds: 3D printed bone tissue scaffolds can mimic the natural skeletal environment to promote bone regeneration and vascularisation, providing a new strategy for sports injury repair.^96^ Bioactive material coatings: by combining advanced biomaterials and gradient porous scaffold design, 3D printing can enhance the biocompatibility of implants, improve implant stability and accelerate recovery from sports injuries (see A in Fig. 4).^97^

For athletes with amputations due to serious sports injuries, 3D printing offers highly personalised and high-performance solutions for prosthetics and rehabilitation supports, customised prosthetics and supports: traditional prosthetics tend to be fixed in size and less comfortable to wear, whereas 3D printing technology allows the customisation of lightweight, high-strength prosthetics and supports according to athletes' physiological characteristics and athletic needs, providing a more natural sporting experience.^94–96^ Bionic materials and mechanical optimisation: additive manufacturing can use advanced materials such as DLC-coated metal alloys CoCrMo and Ti_6_Al_4_V to improve the wear resistance of prostheses and implants, reduce frictional losses and increase longevity. Optimisation of prosthetic contact surfaces: 3D printed CoCrMo and Ti_6_Al_4_V/DLC samples have been found to have low coefficients of friction (CoF) in bioslip environments, which improves implant lubrication, reduces frictional discomfort at the prosthesis–tissue interface, and improves the athlete's experience of using the prosthesis (see C in Fig. 4).^98^

##### 3.2.3.3 3D printing in smart sports monitoring and injury prevention

In competitive sports, prevention and early intervention of sports injuries are equally important, and 3D printing combined with smart sensing technology offers advanced solutions for sports monitoring, injury warning and rehabilitation assessment.^99–101^ Smart wearable devices: 3D printing can be used to produce customised sports monitoring insoles and protective gear, combined with smart sensors to provide gait analysis, posture correction and injury warning.^102^ Patient-centric sensors: by introducing molecular biomarker detection, athletes can be monitored in real time for biosignals such as muscle fatigue and inflammatory responses to optimise sports training and prevent sports injuries (see B in Fig. 4).^103^ Personalised rehabilitation devices: 3D printed rehabilitation supports combined with smart sensors can monitor athletes' joint mobility and muscle recovery in real time and provide personalised rehabilitation guidance to improve rehabilitation efficiency.^104^

#### 3.2.4 Comparative analysis of modern high-tech sports injury materials

With the development of materials science and bioengineering, modern sports injury materials have made breakthroughs in mechanical properties, intelligence, bionic repair and personalised fit. To make the advantages of different types of materials more intuitive, a detailed comparison of different types of modern sports injury materials is presented below (see Table 2). Characteristics of nanomaterials: nanofibre scaffolds, nanoparticle coatings and other materials are high-strength, lightweight and biocompatible, and can be used for ligament injury repair, antimicrobial protective gear, tissue engineering repair and so on. Studies have shown that nanofibre scaffolds promote ligament cell adhesion and proliferation and improve repair efficiency (experimental data: cell proliferation rate increased by 40%, *P* < 0.05). Properties of polymer composites: polymer materials have good mechanical properties, durability and can be used in protective equipment, orthopaedic implants, *etc.* Clinical studies have found that fracture patients with PEEK implants have 30% fewer post-operative complications than with traditional metal implants. 3D Printing material characteristics: 3D printing technology enables the production of personalised protective equipment and implants, optimising biomechanical fit and reducing the risk of secondary injury. 3D printed bone implants combined with a bioactive coating can increase the rate of osteoblastic cell proliferation and promote osseointegration by 25%. 3D printing can also be used to produce bone implants that can be used to repair bone fractures.

**Table 2:** Comparison of different modern high-tech sports injury materials

Material types	Main functions	Advantages	Limitations	Typical applications
Nanomaterials	Enhances tissue repair and intelligently monitors movement data to improve material strength	Lightweight, high strength, antimicrobial properties, increased speed of repair	Higher cost, long term stability and immune rejection needs to be proven	Sports protective gear, artificial ligaments, bone tissue repair
Polymer composites	Provides high toughness support, reduces impact and improves durability of sports equipment	Lightweight and high strength, impact resistant, biocompatible	Manufacturing process is complex, some materials still need to be improved to enhance biodegradability	Sports shoe soles, rehabilitation supports, orthopaedic implants
3D printing materials	Personalised sports protectors and rehabilitation braces to optimise biomechanics	Can be precisely matched to individual needs to improve comfort and rehabilitation	Higher production costs, longer manufacturing time, long-term stability of some materials to be verified	Sports protectors, fracture fixation braces, artificial meniscus

### 3.3 Application scenario analysis of matching sports injury types and materials

In competitive sports, the frequency of sports injuries is high, and the types of injuries in different parts of the body are different. Therefore, the selection of appropriate sports injury materials for different injury types is crucial for improving the protective effect of athletes and shortening the rehabilitation cycle. According to the occurrence mechanism and parts of sports injuries, they can be mainly divided into joint ligament injuries, tendon injuries, fracture injuries and soft tissue injuries, and different types of injuries are suitable for different materials and technologies.

#### 3.3.1 Joint ligament injuries: combined application of polymer composites and nanofibre scaffolds

Ligament injuries occur mainly in the knee (anterior cruciate ligament, medial collateral ligament) and ankle (lateral ligament) and are usually caused by severe twisting or hyperextension, and basketball players' knee joints are subjected to excessive torque during landing, which can lead to ACL strains or even ruptures.^49,64^ Polymer composites (*e.g.* carbon fibre reinforced polymers) have been used in knee and ankle pads to provide good support and stability and to reduce abnormal range of motion of the joints, thus reducing the risk of ligament injuries, while 3D printed personalised pads can be customised according to the biomechanical data of athletes to improve comfort and protective performance.^105^ Nanofibrous scaffolds have been widely used for ligament repair, and silk protein-polycaprolactone (SF/PCL) nanofibrous membranes, which have shown higher cell adhesion and biocompatibility than conventional materials in experimental studies, contribute to the proliferation and regeneration of ligament cells.^60^

#### 3.3.2 Tendon injuries: synergistic effects of bionic hydrogel and nano-reinforcement materials

The tendon connects muscle and bone and is one of the most stressed tissues in high-intensity sports.^106^ Prolonged repetitive use, improper training or external influences (*e.g.* 'tennis elbow’ in tennis players) can lead to injuries such as tendonitis and tendon rupture.^107^ Biomimetic hydrogels can be used for tendon repair, such as polyethylene glycol-gelatin-based hydrogels, which mimic the elastic properties of natural tendons while providing excellent water retention, which aids in the regeneration of damaged tendon tissue.^108^ Nano-reinforced materials (*e.g.* nano-hydroxyapatite, carbon nanotube composites) can be used in artificial tendon implants to improve the mechanical strength of the material, bringing it closer to the loading capacity of natural tendons and reducing the risk of post-operative injury.^109–112^

#### 3.3.3 Fracture injuries: 3D-printed bone scaffolds with smart biomaterials

Fractures are a more serious type of injury in competitive sports, especially in high-impact sports (*e.g.* football, skiing, gymnastics), and common sites include the clavicle, tibia, and carpal bones, *etc.* [3D-printed bone scaffolds combined with bioactive materials (hydroxyapatite-poly(lactic acid) complexes) have been widely used in fracture treatment, which not only have good mechanical strength, but can also promote bone tissue regeneration, which improves the speed of fracture healing.^113–115^ Smart biomaterials (*e.g.* degradable magnesium alloy implants) can gradually degrade *in vivo*, reducing the risk of secondary surgical removal of conventional metal implants, and at the same time the magnesium ions released by them can promote the proliferation of osteoblasts, which is helpful for fracture repair.^116–119^

#### 3.3.4 Soft tissue injuries: combined application of anti-inflammatory nanomaterials and smart sensors

Soft tissue injuries (*e.g.* muscle strains and bruises) are the most common type of injury in athletes, particularly in sports that require explosive force, such as sprinting, long jumping, basketball and rugby.^120,121^ Nano-anti-inflammatory materials (*e.g.* nanosilver, nano-zinc oxide coatings) can be used in sports plasters and cold compresses, which have antimicrobial and anti-inflammatory effects that can effectively reduce inflammatory responses after injury and promote tissue repair.^122–124^ Smart sensors combined with wearable devices (*e.g.* flexible electronic skin) can monitor muscle activity status in real time and provide early warning of injury.^125,126^ Flexible sensors can detect small changes in muscle strain and alert athletes when they are in a high-risk state to reduce the incidence of soft tissue injuries.^127–130^

With the further development of sports biomechanics, smart materials and bioengineering, future materials for sports injuries will be more precise and personalised. Different types of injuries will be matched with more suitable polymer composites, nanomaterials and 3D printing technology, and combined with intelligent monitoring systems to form a more complete system for the protection and treatment of sports injuries. By optimising the mechanical properties of materials, biocompatibility and intelligent detection capabilities, the sports life and competitive level of athletes will be more effectively protected.

#### 3.3.5 Application of smart and intelligent biomaterials in sports injury prevention and rehabilitation

In recent years, the integration of smart textiles and intelligent biomaterials has opened new frontiers in the prevention and rehabilitation of sports injuries.^131^ Smart textiles, which combine flexible, breathable, and skin-friendly fibers with embedded sensing and data-processing capabilities, provide a unique platform for continuous biomechanical monitoring and adaptive protection. The latest studies highlight the potential of smart textiles not only in personalized healthcare—such as physiological signal monitoring, injury diagnosis, and localized therapy—but also in sustainability, through energy harvesting and body temperature regulation.^132^

Building upon these developments, AI-assisted smart sensing systems can be embedded into wearable devices or protective gear to achieve real-time monitoring, risk prediction, and adaptive adjustment of mechanical support. These systems acquire biomechanical data through high-precision pressure sensors, optical devices (*e.g.*, laser interferometers), and wearable biosensors, which are then analyzed *via* deep learning models to predict potential injuries and provide corrective feedback. Furthermore, intelligent repair systems—such as adaptive braces and self-regulating materials—are being explored to automatically adjust their stiffness and support levels in response to detected fatigue or injury signals.^133–135^

This convergence of biomaterials, artificial intelligence (AI), and Internet of Things (IoT) technologies represents a major leap toward personalized, real-time, and intelligent sports injury management, paving the way for next-generation wearable healthcare systems that integrate monitoring, prevention, and adaptive rehabilitation.

## 4. Summary, future trends and challenges and recommendations

### 4.1 Summary

This study thoroughly explores the application of sports injury materials in competitive sports, covering the role of traditional materials, polymer composites, nanomaterials and 3D printed materials in the prevention, treatment and rehabilitation of sports injuries. It is found that traditional sports injury materials (such as protective gear, cold and hot dressings, elastic bandages, *etc.*) have basic protective functions but have limitations such as high stiffness, poor individual adaptability and single function. Polymer composites (*e.g.* polyetheretherketone PEEK, graphene composites, *etc.*) are widely used in sports protective equipment, rehabilitation stents and orthopaedic implants due to their characteristics of high strength, light weight and durability, which have significantly improved the effect of sports injury protection. Nanomaterials (*e.g.* nanofibre scaffolds, biomimetic photothermal nanomaterials, nanocoatings, *etc.*) show the potential of self-repairing ability, intelligent response and precise targeted therapy, which can accelerate the repair of damaged tissues and play an important role in the fields of sports injury detection and smart drug release. In addition, 3D printed materials have greatly improved the precision of sports injury prevention and treatment through personalisation, highly bionic structure and flexible sensing technology, enabling individualised sports injury protection. The study also found that the combination of multifunctional sports injury materials is one of the future trends. For example, the combination of nanotechnology and 3D printing can create intelligent bionic tissue engineering scaffolds for the repair of bone, cartilage and ligament injuries; the use of smart wearable devices combined with flexible sensing materials can provide real-time monitoring and feedback of sports injuries and improve the safety of athletes' training. The integration of artificial intelligence and biomaterials will enable more accurate injury prediction and personalised rehabilitation programmes, reducing the incidence of injury in competitive sports and speeding up the recovery process.^136–138^

### 4.2 Future development trends

In the future, the development of sports injury materials will move towards intelligence, personalisation, biomimicry and sustainability, and further optimise their feasibility and cost-effectiveness in practical applications.

#### 4.2.1 Personalisation: 3D printing meets precision medicine

Protection and rehabilitation from sports injuries need to be optimised for individual characteristics, and the combination of 3D printing and biomaterials science will drive the development of personalised and customised protective gear, orthopaedic implants and rehabilitation scaffolds. For example, 3D printed personalised protective gear can be accurately modelled according to an athlete's bone structure, training habits and force distribution to achieve a more biomechanically correct protective effect. The implementation path includes: data input: based on CT scanning, MRI imaging technology to obtain the athlete's bone and joint data, combined with sports biomechanics modelling, accurate analysis of individual injury risk areas. Intelligent manufacturing: combined with high-resolution 3D printing, personalised protective equipment or rehabilitation braces can be rapidly produced. For example, nanocomposites + flexible printing technology can be used to produce high-strength, lightweight protective gear to improve comfort and stability. Cost optimisation: at present, 3D printing has the problems of high cost and long production cycle, in the future, it can reduce the cost by multi-material co-printing, improve the printing speed, optimise the material formula, *etc.*, so that it has the possibility of large-scale application.

#### 4.2.2 Biomimetic materials: mimic human tissue to improve repair capabilities

In the future, biomimetic materials will play a key role in tissue repair and sports injury treatment. For example, biomimetic photothermal nanomaterials can make use of the photothermal conversion effect to promote blood circulation at the injury site and accelerate cell regeneration; smart hydrogels can mimic the structure of natural cartilage to improve the treatment efficiency of sports injuries. The realisation path includes: material optimisation: developing biomaterials that are closer to the mechanical properties of natural tissues, such as smart hydrogel (for cartilage repair) and biomimetic fibres (for ligament reconstruction), in order to enhance biocompatibility and repair effects. Clinical translation: use animal models and long-term biological experiments to evaluate the stability, immunocompatibility and degradation properties of new materials *in vivo* to ensure their safety and efficacy. Application example: tissue-engineered scaffolds based on nano-biomaterials can be applied to repair cartilage injuries in the knee joint and improve the speed of post-operative rehabilitation for athletes.

#### 4.2.3 Toward intelligent, sustainable, and self-powered biomaterials

Despite remarkable progress in smart materials and wearable monitoring systems, energy sustainability remains one of the main challenges for continuous, real-time operation. Traditional batteries are limited by short lifespan and frequent charging needs, which restrict the autonomy of wearable systems. To address these issues, researchers have developed self-powered nanogenerators, such as triboelectric (TENG) and piezoelectric nanogenerators (PENG), that harvest biomechanical energy from natural human motions (*e.g.*, walking, running, or muscle contraction) to achieve wireless and autonomous energy supply.

Recent advances in hybrid energy materials—including supercapacitors, conductive polymers, and MXene-based composites—have significantly improved the energy conversion efficiency and storage capacity of such devices. These flexible and biocompatible systems pave the way for sustainable, self-powered motion-monitoring platforms capable of real-time sensing, adaptive feedback, and long-term operation without the need for external charging sources.

With increasing global concern for environmental protection and sustainability, future sports injury materials will focus more on degradability, biocompatibility and low energy production. For example, natural polymers (*e.g.* chitosan, biodegradable polylactic acid) will be used to produce biodegradable protective gear or implants to reduce environmental impact. Ways to achieve this include low carbon production: using 3D printing + biodegradable biomaterials to reduce material waste and optimise the manufacturing process to reduce carbon emissions. Long-term stability: ensuring that degradable materials have sufficient mechanical properties and degradation rate in the injury repair process to meet the needs of clinical applications.

In the future, the trend of intelligent, personalised, biomimetic and sustainable development of sports injury materials will significantly improve the protective effect, rehabilitation efficiency and comfort of athletes. Through the intelligent sensing system + big data analysis + 3D printing of personalised protective gear, can provide more accurate sports injury prevention and treatment programme. Meanwhile, breakthroughs in biomimetic materials will accelerate injury repair, while the application of sustainable materials will contribute to the development of environmentally friendly competitive sports. With the integration of multidisciplinary technologies, sports injury materials will play a more important role in competitive sports in the future, providing athletes with safer, more efficient and intelligent protection and rehabilitation solutions.

### 4.3 Challenges and limitations

Despite remarkable progress in the development of smart biomaterials and wearable technologies for sports injury prevention, several challenges remain before these systems can be fully implemented in real-world environments. These challenges involve long-term stability and biocompatibility, intelligence and integration, manufacturing scalability and cost, and clinical translation and personalization.

#### 4.3.1 Long-term stability and biocompatibility

Many emerging materials—such as nanocomposites, biomimetic hydrogels, and polymer blends—demonstrate excellent performance in laboratory or short-term animal studies. However, their long-term mechanical durability, degradation behavior, and biosafety under real physiological conditions remain unclear. For instance, nanomaterials may gradually degrade *in vivo*, and their degradation by-products could trigger inflammatory or immune responses. Therefore, the evaluation of biodegradability, histocompatibility, and immune adaptation through extended animal testing and multi-center clinical trials is essential. Surface modification techniques (*e.g.*, anti-inflammatory coatings or bioactive functionalization) may further enhance the immunocompatibility of next-generation biomaterials.

#### 4.3.2 Intelligence, integration, and energy supply

Smart sensing materials and wearable systems hold great potential for real-time monitoring and rehabilitation guidance, yet most devices remain at the prototype validation stage. Challenges such as sensor accuracy, data transmission stability, and durability limit their performance in high-intensity sports settings. Furthermore, self-powered nanogenerators often provide low voltage outputs, which restrict long-term operation. Future research should focus on optimizing sensor sensitivity, wireless communication, and hybrid energy systems, integrating materials science, biomedical engineering, and artificial intelligence to achieve reliable, real-time feedback.

#### 4.3.3 Manufacturing scalability and personalization

Although 3D printing enables personalized protective and rehabilitative devices, its widespread application is restricted by high costs, long printing cycles, and limited material options. High-precision bioprinting, for instance, can fabricate complex bioscaffolds for soft tissue repair but remains too expensive and time-consuming for rapid clinical deployment. Future strategies should focus on improving printing efficiency (*e.g.*, high-speed laser printing), developing high-strength bio-inks, and reducing material costs through multi-material co-printing. The integration of 3D printing with AI-driven data analytics could further allow athlete-specific design based on individual biomechanics and injury profiles.

#### 4.3.4 Clinical translation and multidisciplinary collaboration

Finally, sports injuries are influenced by individual physiology, training intensity, and environmental factors. Relying solely on materials innovation is insufficient. The future lies in integrating big data analytics, AI-based prediction models, and biomechanical simulation to guide training and prevent injuries. Close collaboration among materials scientists, sports physicians, and engineers will be crucial to transform laboratory prototypes into clinically viable and commercially scalable solutions.

## Conflicts of interest

There are no conflicts to declare.

## Note added after first publication

This article replaces the version published on 7th January 2026, with updated versions of the Graphical Abstract image and Fig. 1.

## Data Availability

The data supporting the findings of this study are publicly available in the Science Data Bank. The dataset can be accessed *via* the following DOI: [https://doi.org/10.57760/sciencedb.34588].
